# Significance of MALAT1 long non-coding RNA and miR-20a-5p in regulating epithelial mesenchymal transition in luminal breast cancer patients

**DOI:** 10.1186/s43046-026-00339-w

**Published:** 2026-02-02

**Authors:** Gehad Tarek, Manar Fouda, Mohamed Omran, Gehan Safwat, Mahmoud Kamel, Abdel Hady Abdel Wahab

**Affiliations:** 1 Department of Chemistry, Faculty of Science, Helwan University, Cairo, Egypt; 2 Faculty of Biotechnology, October University of Modern Sciences and Arts, Cairo, Egypt; 3 Department of Clinical Pathology,, National Cancer Institute, Cairo University, Cairo, Egypt; 4 Baheya Foundations for Early Detection and Treatment of Breast Cancer, Giza, Egypt; 5 Department of Cancer Biology, National Cancer Institute, Cairo University, Cairo, Egypt

**Keywords:** Breast cancer, Non-coding RNA, MALAT1, MicroRNA, miR-20a-5p, Luminal subtype

## Abstract

**Background:**

Luminal breast cancer (LBC) is the most common subtype of breast cancer affecting women worldwide. Although luminal breast cancer typically has a better prognosis, it mostly responds poorly to neoadjuvant chemotherapy. Non-coding RNAs, especially long non-coding RNAs and microRNAs are crucial in regulating biological processes that contribute to breast cancer development. MALAT1, a long non-coding RNA, is pivotal in the progression of breast cancer. Epithelial-mesenchymal transition (EMT) is critical for cell movement during embryonic development. Clarifying this role could pave various avenues for developing innovative strategies for combating this subtype of malignancy. The present study aimed to investigate the expression profiles and clinical relevance of MALAT1 level and EMT-related miRNAs (miR-17-5p, miR-20a-5p, miR-93-5p, miR-135b-5p, and miR-146a-5p) alongside EMT markers (E-cadherin, N-cadherin, vimentin, fibronectin, twist, SNAI1, Slug, ZEB1, and ZEB2) in LBC patients.

**Methods:**

Fresh tissues were collected from fifty patients and twenty noncancerous controls. Differential expression of the markers was evaluated using qRT-PCR assay. Spearman Rho test assessed the relationship between the expression levels. Linear regression test evaluated the correlation between the parameters and various clinico-pathological features.

**Results:**

Our results revealed an overall upregulation of MALAT1 in breast cancer tissues although this increase did not reach statistical significance. Overexpression of miR-20a-5p, miR-135b, and ZEB2 was reported, whereas miR146a-5p, ZEB1 and Vimentin levels were suppressed. Correlation analysis demonstrated that miR-20a-5p was positively correlated with SNAI1, E-cadherin, N-cadherin and Slug also it was significantly associated with family history and tumor laterality.

**Conclusions:**

Our findings suggest that miR-20a-5p plays an oncogenic role in luminal breast cancer by promoting EMT, while MALAT1 may contribute to disease progression through indirect regulatory mechanisms. Finally, MALAT1 and miR-20a-5p might serve as potential therapeutic and prognostic targets in LBC.

## Background

Globally, breast cancer accounts for approximately 2.3 million new cases annually, representing a major cause of cancer-related morbidity and mortality among women [1]. It is a highly heterogeneous disease that can be divided into numerous subtypes depending on molecular and clinicopathological characteristics, such as the presence of hormone receptors (estrogen and progesterone receptors) and the status of human epidermal growth factor receptor 2 (HER2) [2]. Luminal breast cancer (LBC), which is positive for estrogen and/or progesterone receptors, is the most common type of breast cancer around the world. It makes up most of the cases in Egypt and other regions globally [3]. Although it usually has a better prognosis, luminal breast cancer tends to have less response to neoadjuvant chemotherapy given before surgery compared to other types like HER2-enriched and triple-negative breast cancers [4]. Since the goal of neoadjuvant chemotherapy is to shrink tumors and check how well they respond to treatment, it is important to predict how luminal breast cancer will respond. This helps prevent patients from getting unnecessary chemotherapy, lowers the risk of side effects, and allows for more personalized treatment plans for those who may not benefit from it^5^. For these reasons, this study focuses on luminal breast cancer to tackle this ongoing challenge in clinical practice. It aims to find biomarkers that can help make better decisions about treatment for this common but difficult type of cancer.

It has been demonstrated that the majority of the human genome can be transcribed into RNA, but only a small fraction of it does not generate proteins, keeping the rest of the genome non-coding RNA [6]. There are various kinds of non-coding RNAs, with particular emphasis on long non-coding RNAs (lncRNAs) and microRNAs (miRNAs), which have been extensively studied due to their crucial role in tumor drug resistance mechanisms, both through single actions and their interplay [7]. LncRNAs are a group of RNA molecules that play crucial regulatory roles in various cellular processes. These molecules are expressed in many different tissues and play important roles in controlling the activity of genes. LncRNAs undergo different processes of formation than mRNAs, and they possess unique locations and activities within cells [8]. It is now widely accepted that lncRNAs can influence gene expression by engaging chromatin-modifying complexes, serving as scaffolding for protein‒protein interactions, or by directly binding to target mRNA molecules [9]. The aberrant regulation of long non-coding RNAs has been linked to a range of human diseases, including cancer [10].

MALAT1, also known as Metastasis-Associated Lung Adenocarcinoma Transcript 1, is a widely recognized long noncoding RNA and is a highly conserved nuclear lncRNA transcribed from human chromosome 11q13. Initially, it was recognized as a transcript that is abundantly expressed in metastatic lung cancer cells [11]. MALAT1 is widely present in normal tissues and plays a significant role in multiple cellular activities, such as controlling gene expression, regulating alternative splicing, and organizing the nucleus [12]. MALAT1 has been proved to be highly expressed in numerous human cancers, including breast [13]. High MALAT1 expression has been reported in breast cancer tissues and is related with poor prognosis [14]. Moreover, silencing MALAT1 promoted the apoptosis of breast cancer MCF7 cells resistant to adriamycin and taxanes [15]. Additionally, MALAT1 is highly expressed in exosomal breast cancer cells and enhances metastasis and drug resistance by controlling the miR-1-3p/VASP/Rap1 signaling axis [16].

Previous studies have indicated a direct and effective link between MALAT1 and the epithelial-mesenchymal transition (EMT) process. MALAT1 enhances EMT, which is connected to how cancer cells resist treatment and metastasis [17]. Specifically, MALAT1 influences EMT by controlling important proteins that control gene activity and by sponging the microRNAs, which usually help regulate genes. This makes cancer cells more likely to move and spread [18].

Therefore the present study aimed to evaluate the expression and clinical significance of MALAT1 in luminal breast cancer in Egyptian patients by controlling the gene expression of pertinent miRNAs that play a significant role in the EMT pathway, which contributes to metastasis and chemoresistance.

## Methods

### Specimen collection

This study was approved by the Baheya-Research Ethical Committee, Baheya Foundation for Early Detection & Treatment of Breast Cancer, Egypt (IRB#00012829). Fresh tissue specimens were collected from fifty LBC patients treated at Baheya Hospital between February 2022 and December 2023 using the core needle biopsy technique. Non-cancerous tissues were also collected from twenty patients as controls. All tissues were obtained before starting any kind of treatment. One part of the tissue collected was preserved in buffered formalin for histopathological analysis, and the other part was immediately preserved at -80 °C for subsequent analysis. Various clinico-pathological data were collected and are summarized in Table 1. Informed consent was obtained from all participants in compliance with the code of ethics of the World Medical Association (Declaration of Helsinki).

**Table 1:** Clinico-pathological features for the luminal breast cancer patients

Variables	Categories	N (%)
Age (years)	mean ±SD	58±12.5
Menopause	Yes	36 (72)
	No	14 (28)
Family history	Yes	26 (52)
	No	24 (48)
Luminal subtype	A	48 (94)
	B	2 (6)
Laterality	Right	36 (72)
	Left	14 (28)
Histology	Ductal carcinoma	46 (92)
	Lobular carcinoma	4 (8)
Histological grade	G1	4 (8)
	G2	37 (74)
	G3	9 (18)
Hormonal status	ER+	50 (100)
	PR+	46 (92)
Tumor size (cm)	<5	41 (82)
	≥5	9 (18)
TNM stage	Stage I-II	29 (58)
	Stage III-V	21 (42)
Lymph node metastasis	Negative	16 (32)
	Positive	34 (68)
Distant metastasis	Absent	50 (100)
	Present	0 (0)

### Quantitative real-time polymerase chain reaction (qRT‒PCR)

Using the miRNeasy^®^ Mini Kit (Qiagen, Germany) in accordance with the manufacturer’s instructions, highly purified total RNA, including miRNA, was isolated from the collected frozen tissue specimens. The RNA concentration was quantified using a NanoDrop™ One spectrophotometer (ThermoFisher Scientific, USA). One microgram of the isolated RNA was used for synthesis of complementary DNA (cDNA) using a miScript II RNA reverse transcription kit (Qiagen, Germany) according to the supplied protocol. The cDNA product was diluted 5 times; 2 µl was used for PCR. The relative expression levels of MALAT1 lncRNA, miRNAs (miR-17-5p, miR-20a-5p, miR93-5p, and miR-146a-5p) and EMT markers (E-cadherin, N-cadherin, Vimentin, Fibronectin, Twist, SNAI1, Slug, ZEB1, and ZEB2) were determined using *PerfectStart*^®^ Green qPCR SuperMix (TransGen Biotech Co., Ltd., Beijing, China). Primer sequences were synthesized and obtained from Eurofins Genomics (GmbH, Germany). The assay was performed on a ViiA7 real-time PCR system (Applied Biosystems, Foster City, CA, USA). The thermocycling conditions were as follows: 95 °C for 15 min, followed by 40 cycles of 95 °C for 20 s and 60 °C for 60 s. The samples were performed in duplicate. The relative expression was conducted using the 2^−∆∆Ct^ approach after normalization to RNU6, and GAPDH was used as an endogenous control (Livak and Schmittgen) [19].

### Statistical analysis

IBM SPSS statistics v26.0 software (SPSS Inc., Chicago, Ull, USA) was used for data analysis. All values are expressed as the median ± IQR. Comparisons between two groups were conducted using the Mann‒Whitney test. Spearman’s Rho correlation analysis was adopted for assessing the relationship between two different parameters measured. A logestic regression analysis was performed to determine the correlation between MALAT1 and miRNA levels with various clinic-pathological features of LBC patients. Differences with a p value < 0.05 were considered to be statistically significant.

## Results

### Differential expression of MALAT1 and MiRNAs in LBC patients

**Fig. 1:**
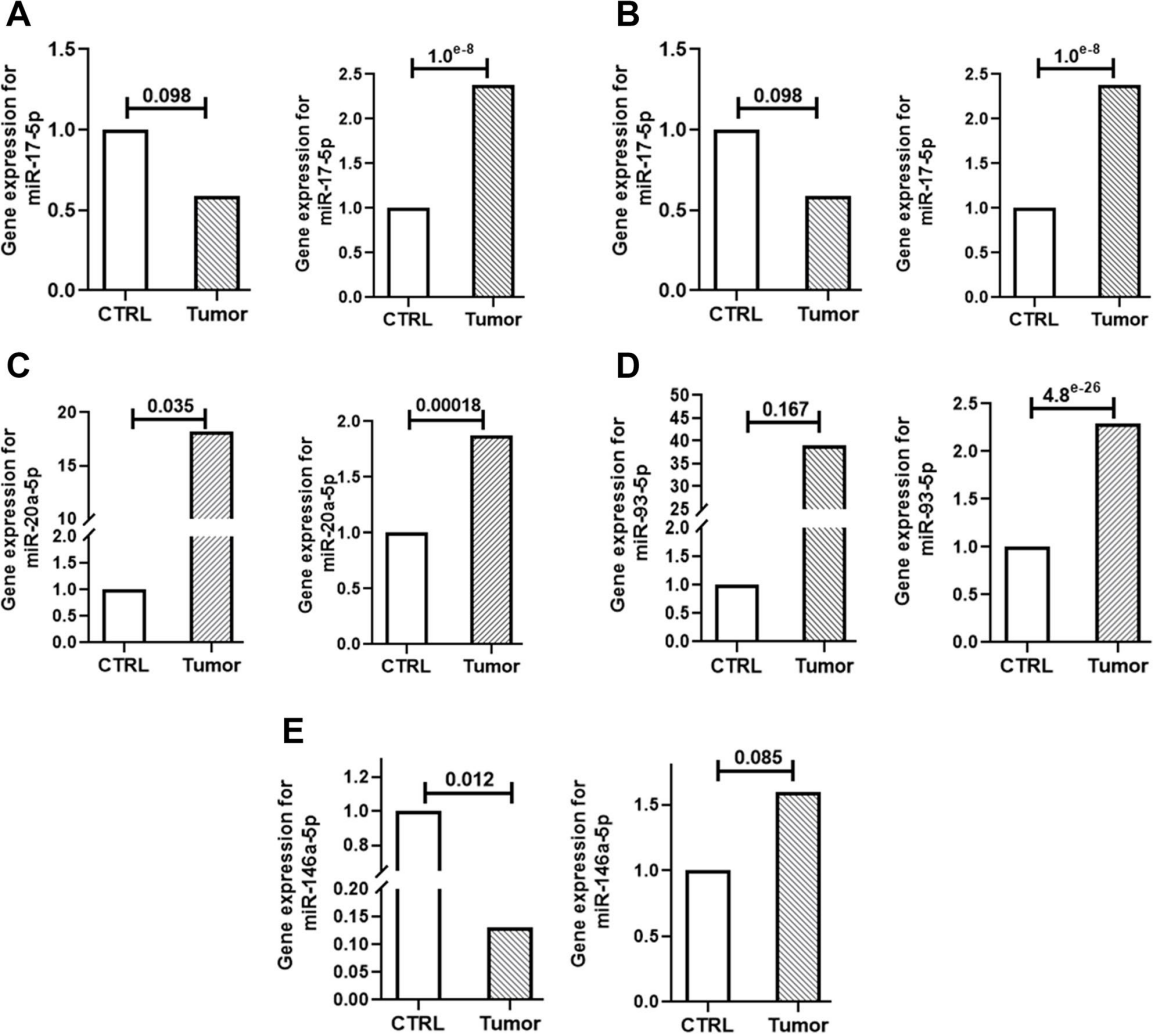
Expression levels of MALAT1 (**a**), miR-17-5p (**b**), miR-20a-5p (**c**), miR-93-5p (**d**), and miR-146a-5p (**e**) in tissues from luminal breast cancer patients from the present study and expression levels from the ENCORI database. *P* values < 0.05 is considered statistically significant

To investigate the importance of lncRNA-MALAT1 in regulating the mechanism of EMT in the progression of LBC as a ceRNA, the expression levels of lncRNA-MALAT1 and a set of related miRNAs (miR-17-5p, miR-20a-5p, miR93-5p, and miR-146a-5p) were determined in tissues from 50 breast cancer patients as well as 20 non-cancerous tissues as controls using qRT‒PCR. MALAT1 expression was greater than threefold in cancerous tissues than in non-cancerous tissues (FC = 3.302); however, this difference was not statistically significant (*p* = 0.979). We investigated the data for the miRNA set evaluated and observed that there were substantial changes in only one of them (miR-20a-p), showing significant increases of 18.18-fold (*p* = 0.035). The expression level of miR-146-5p was shown to be approximately 7.33-fold lower in cancerous tissues than in non-cancerous tissues (*p* = 0.012). On the other hand, an increase in the level of miR-93-5p was detected, reaching more than 38.975-fold; nevertheless, this increase was not statistically significant (*p* = 0.167). Finally, there was no noticeable significant change in the level of miR-17-5p between the two groups analyzed, as the fold change was approximately 0.59 (*p* = 0.098). The ENCORI data revealed a substantial increase in the expression of the MALAT1 gene in LBC tissues compared to that in normal control tissues, but this increase was not significant (FC = 3.49, *p* = 0.095). Significant increase in the expression of miR-20a-p (FC = 1.88, *p* = 0.00018), miR-93-5p (FC = 2.29, *p* = 4.8^e − 26^), and miR-17-5p (FC = 2.38, *p* = 1.0^e − 8^) were detected, but no significant change in the expression of miR-146-5p (FC = 1.60, *p* = 0.085) was detected. Figure 1 (A-E) shows the expression level for MALAT1, and different miRNAs evaluated in the current study as well as data analyzed from ENCORI database.

### Differential expression of EMT markers in LBC patients

**Fig. 2:**
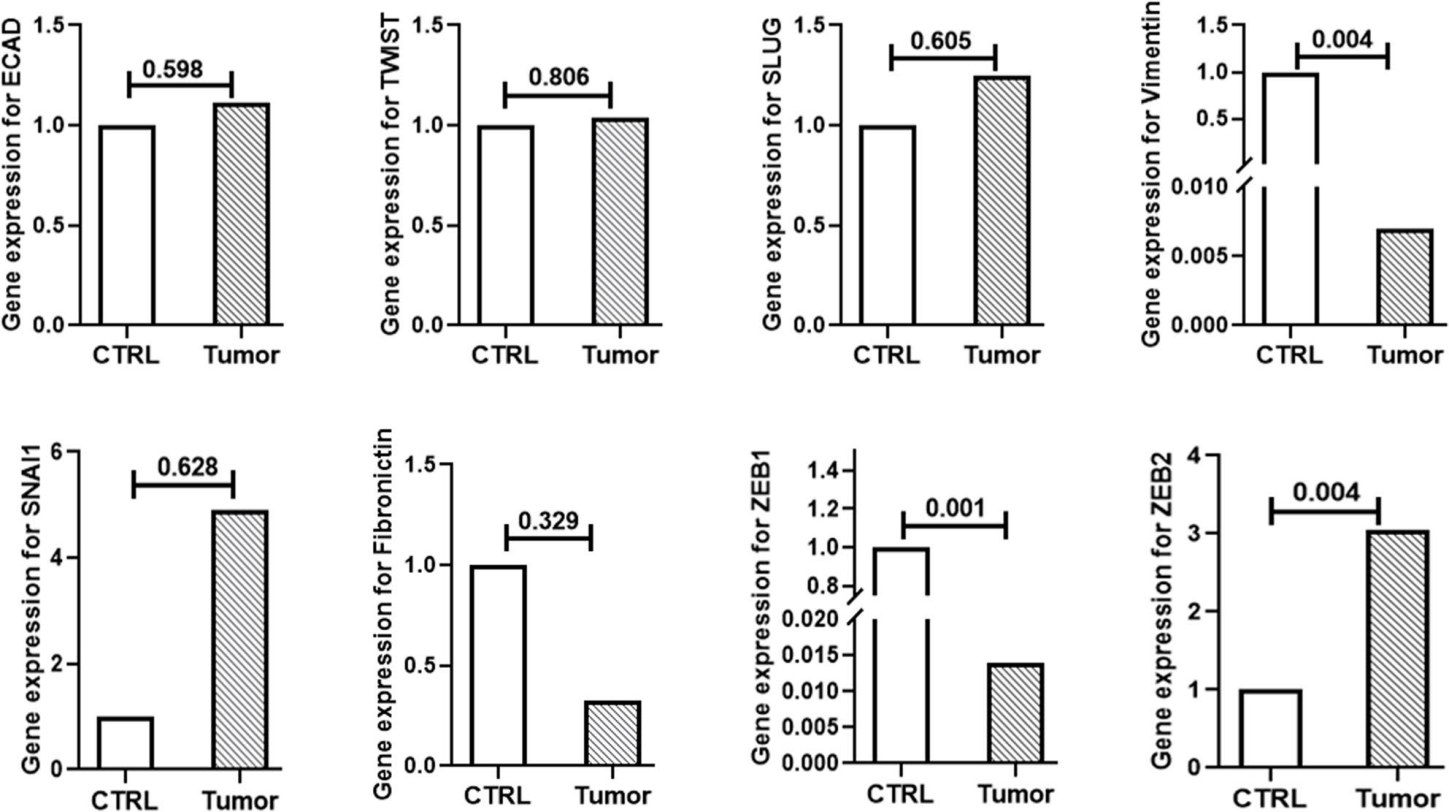
Expression levels of EMT markers including SNAI1, E-Cadherin, N-Cadherin, Twist, Vimentin, Fibronectin, ZEB1, ZEB2 and Slug in tissue from luminal breast cancer patients compared with normal subjects. *P* values < 0.05 is considered statistically significant

EMT plays a crucial role in the progression and development of cancer cells in breast tissue. To assess this role, the gene expression of a set of EMT markers, including E-cadherin, N-cadherin, Vimentin, Fibronectin, Twist, SNAI1, Slug, ZEB1, and ZEB2, was determined via qRT‒PCR. The current findings revealed that ZEB2 (FC = 3.05, *p* = 0.004) was significantly upregulated in tumor tissues compared with that in non-tumor tissues. Moreover, ZEB1 (FC = 0.013, *p* = 0.001) and Vimentin (FC = 0.007, *p* = 0.004) were significantly suppressed in tumor tissues than in non-tumor tissues. None of the other genes investigated demonstrated any substantial deregulation, as indicated in Fig. 2.

### miR-20a-5p is positively correlated with EMT markers

**Fig. 3:**
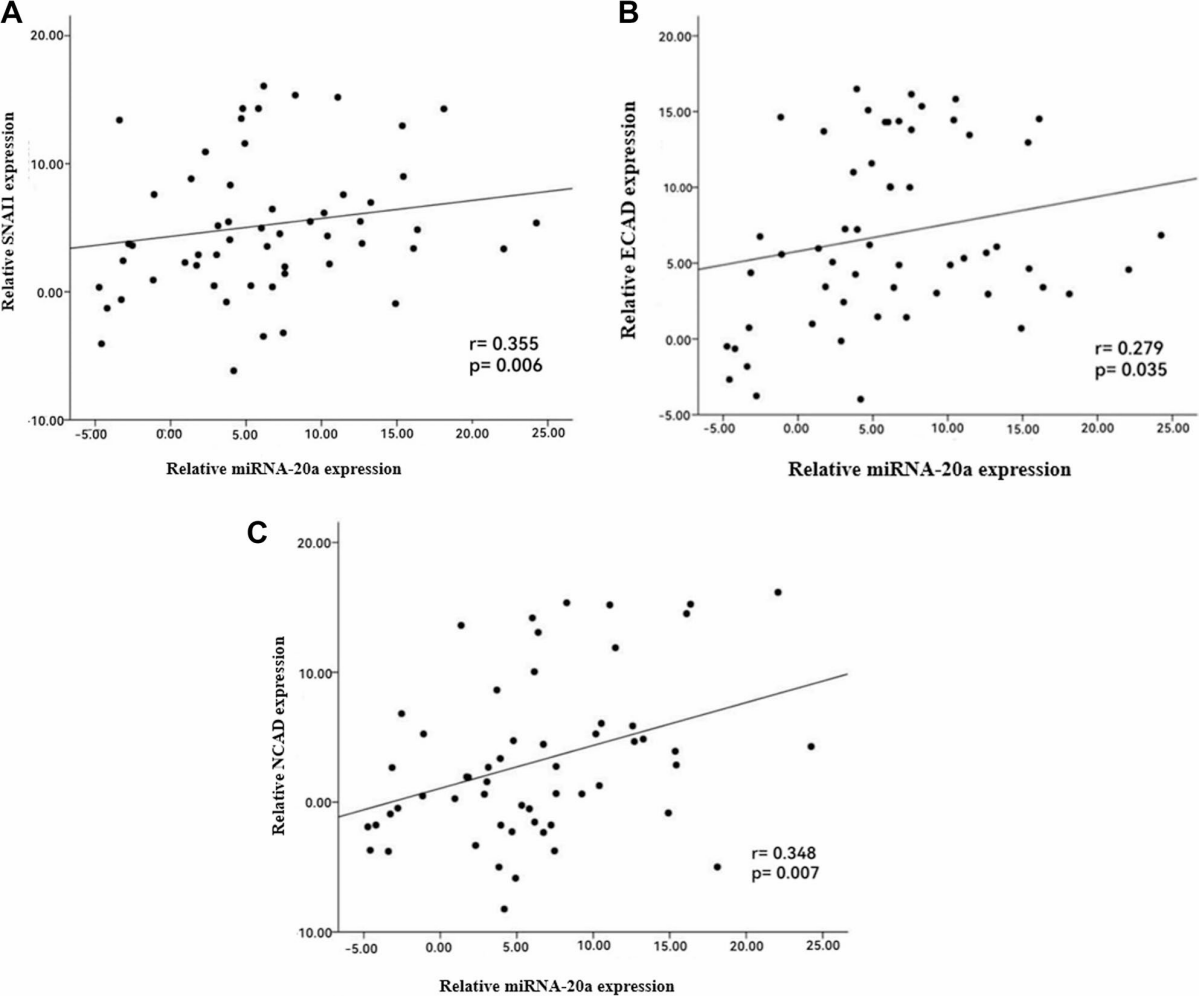
Correlation analysis between expression level of miR-20a-5p with each of SNAI1 (**a**), E-Cadherin (**b**), N-cadherin (**c**), and Slug (**d**) in tissue from luminal breast cancer patients. *P* values < 0.05 is considered statistically significant

To determine whether there is an interaction or association between the impact of the MALAT1 level and either of the microRNAs on the EMT process, we applied Spearman’s Rho correlation test. The results demonstrated that there was no significant association between the levels of MALAT1 and the levels of any of the genes analyzed in this study. On the other hand, miR-20a-5p exhibited a substantial correlation with SNAI1 (*r* = 0.355, *p* = 0.0067), E-cadherin (*r* = 0.279, *p* = 0.0351), N-cadherin (*r* = 0.348, *p* = 0.0078), and Slug (*r* = 0.283, *p* = 0.032), as demonstrated in Fig. 3 (A-D). These results suggest that miR-20a-5p plays a significant role in regulating the EMT process in LBC.

### Correlation between MALAT1 and MiRs expression level with various clinico-pathological features

**Fig. 4:**
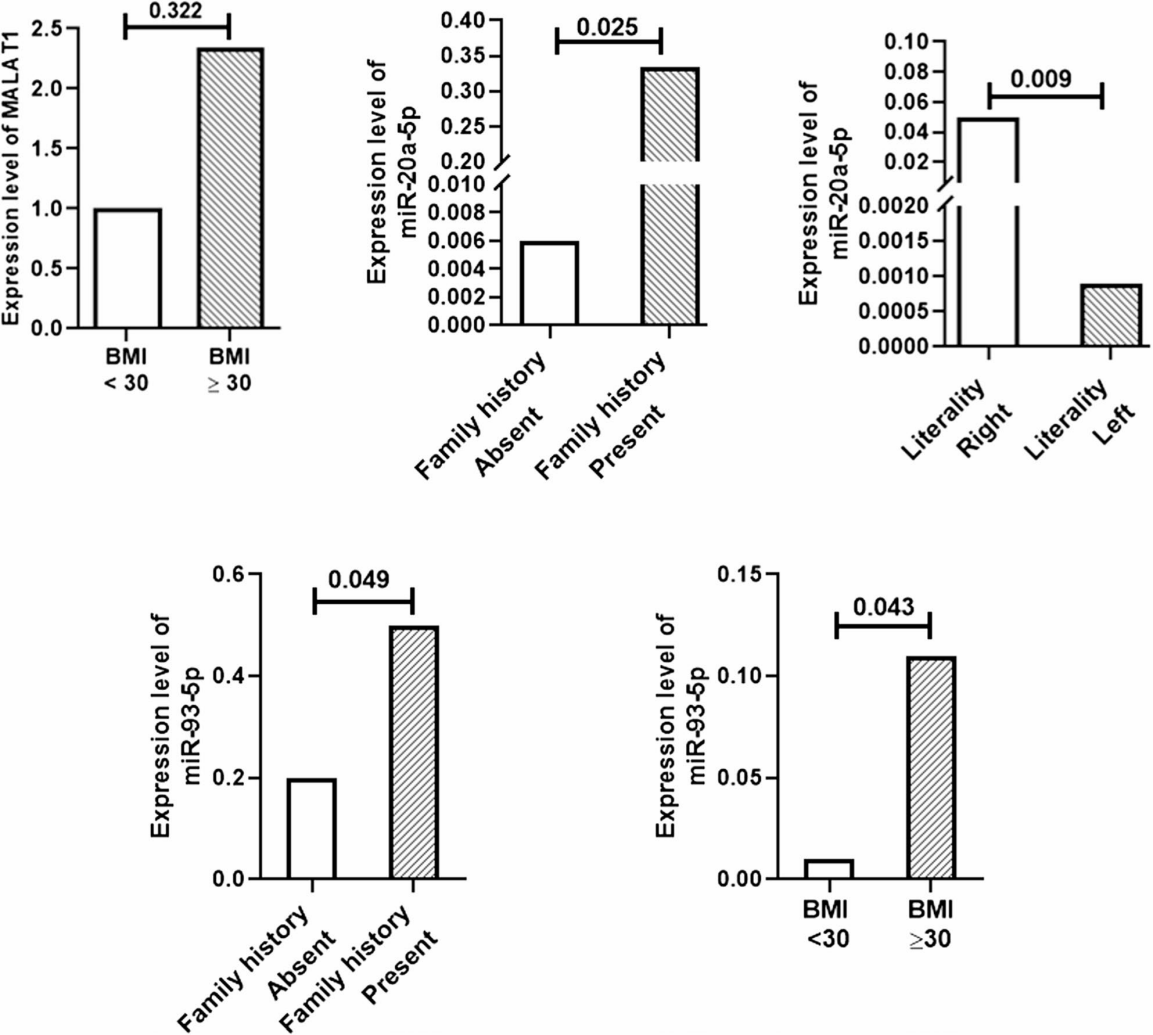
Correlation using linear regression analysis between expression levels of MALAT1 with BMI, miR-20a-5p with family history, miR-20a-5p with laterality, miR-93-5p with BMI, and miR-93-5p with family history in tissue from luminal breast cancer patients. P values < 0.05 is considered statistically significant

In this study, logistic regression analysis was used to evaluate the correlations between the expression levels of MALAT1 as well as miRs in breast tumor tissues with the existing clinico-pathological variables, such as age, lateral relationships, tumor size, body mass index (BMI), and lymph node status. Table 2 indicates that although there was a correlation between the expression of MALAT1 and high BMI patients (OR = 2.5 (0.409–15.893), this correlation did not achieve its significant value (*p* = 0.322). Its expression level in high BMI was lower than that of lower BMI by about 2.31 times. Table 3 showed non-significant correlation between miR-17-5p with any of the variables examined. Table 4 demonstrated a highly significant correlation OR = 8.850 (1.310–59.68) between the patients’ family history and the level of miR-20a-5p. Patients with family history demonstrated high level of miR-20a-5p compared with patients without family history (FC = 1.78). Furthermore, compared to patients with left-sided breast cancer, those with right-sided breast cancer had a substantially higher level of miR-20a-5p (OR = 9.067, (1.624-47.00) by about 3.9 folds. As shown in Table 5, there was a strong correlation found for miR-93-5p between its level and each of high BMI patient (OR = 6.786 (1.062–43.360) and the presence of a family history (OR = 4.875, 1.008–23.568). expression level of miR-135b-5p exhibited negative significant correlation with age of patients [OR = 0.125 (0.017–0.924)] as shown in Table 6. Non-significant correlation between miR146a-5p with either of the clinico-pathological features was observed (Table 7). Figure 4 represents some of the significant correlation between the studies genes with various clinic-pathological data.

**Table 2:** Logistic regression analysis in MALAT1 expression level with odds ratio and 95% CI for various clinico-pathological variables in breast cancer

Variables	β	SE	O	p- value	95%CI	
					Lower	Upper
Age	-0.598	0.861	0.550	0.487	0.102	2.972
Lymph node	1.163	0.801	3.200	0.146	0.666	15.381
BMI	0.916	0.924	2.500	0.322	0.666	15.293
Family history	-0.288	0.726	0.750	0.692	0.181	3.115
Laterality	-0.470	0.725	0.625	0.517	0.151	2.586
Tumor grade	-0.916	0.907	0.400	0.321	0.070	2.450
Tumor size	0.241	0.814	1.273	0.767	0.258	6.273

*β*, regression coefficient; *SE*, standard error; *OR*, odds ratio; *CI*, confidence interval; *BMI*, body mass index

**Table 3:** Logistic regression analysis in miR-17-5p expression level with odds ratio and 95% CI for various clinico-pathological variables in breast cancer

Variables	β	SE	O	p- value	95%CI	
					Lower	Upper
Age	0.588	1.176	1.800	0.617	0.180	8.047
Lymph node	0.446	0.929	1.563	0.631	0.253	9.646
BMI	0.762	0.925	2.143	0.410	0.350	13.121
Family history	1.946	1.150	7.000	0.090	0.736	66.614
Laterality	0.182	0.864	1.200	0.833	0.221	6.521
Tumor grade	-0.588	1.176	0.555	0.617	0.055	5.570
Tumor size	0.288	0.957	1.333	0.764	0.204	8.708

*β*, regression coefficient; *SE*, standard error; *OR*, odds ratio; *CI*, confidence interval; *BMI*, body mass index

**Table 4:** Logistic regression analysis in miR-20a-5p expression level with odds ratio and 95% CI for various clinico-pathological variables in breast cancer

Variables	β	SE	O	p- value	95%CI	
					Lower	Upper
Age	-0.523	0.874	0.593	0.549	0.107	3.286
Lymph node	-0.799	0.772	0.450	0.301	0.099	2.043
BMI	0.280	0.933	1.324	0.764	0.213	8.235
Family history	2.180	0.974	8.850	0.025	1.310	59.68
Laterality	2.205	0.847	9.067	0.009	1.624	47.00
Tumor grade	0.280	0.933	1.323	0.764	0.213	8.226
Tumor size	0.243	0.847	1.275	0.774	0.242	6.704

*β*, regression coefficient; *SE*, standard error; *OR*, odds ratio; *CI*, confidence interval; *BMI*, body mass index

**Table 5:** Logistic regression analysis in miR-93-5p expression level with odds ratio and 95% CI for various clinico-pathological variables in breast cancer

Variables	β	SE	O	p- value	95%CI	
					Lower	Upper
Age	-0.446	0.929	0.640	0.631	0.104	3.951
Lymph node	0.580	0.759	1.786	0.445	0.403	7.906
BMI	1.915	0.946	6.786	0.043	1.062	43.360
Family history	1.584	0.804	4.875	0.049	1.008	23.568
Laterality	1.705	0.889	5.500	0.055	0.962	31.431
Tumor grade	0.446	0.929	1.563	0.631	0.253	9.640
Tumor size	-0.754	0.827	1.563	0.362	0.093	2.380

*β*, regression coefficient; *SE*, standard error; *OR,* odds ratio; *CI*, confidence interval; *BMI*, body mass index

**Table 6:** Logistic regression analysis in miR-135b-5p expression level with odds ratio and 95% CI for various clinico-pathological variables in breast cancer

Variables	β	SE	O	p- value	95%CI	
					Lower	Upper
Age	-2.079	1.021	0.125	0.042	0.017	0.924
Lymph node	-0.531	0.909	0.588	0.588	0.099	3.491
BMI	0.357	1.189	1.429	0.764	0.139	14.695
Family history	-0.693	0.913	0.500	0.448	0.084	2.992
Laterality	1.224	0.953	3.400	0.199	0.525	22.027
Tumor grade	0.788	0.998	2.200	0.429	0.311	15.548
Tumor size	0.560	0.982	1.750	0.569	0.255	11.992

*β*, regression coefficient; *SE*, standard error; *OR*, odds ratio; *CI*, confidence interval; *BMI,* body mass index

**Table 7:** Logistic regression analysis in miR-146a-5p expression level with odds ratio and 95% CI for various clinico-pathological variables in breast cancer

Variables	β	SE	O	p- value	95%CI	
					Lower	Upper
Age	-1.946	1.150	0.143	0.090	0.015	1.360
Lymph node	-0.470	0.725	0.625	0.517	0.151	2.586
BMI	-0.134	0.859	0.875	0.876	0.162	4.713
Family history	-0.241	0.731	0.786	0.741	0.188	3.290
Laterality	1.386	0.799	3.998	0.083	0.835	19.162
Tumor grade	-0.598	0.861	0.550	0.487	0.102	2.792
Tumor size	-0.241	0.814	0.786	0.767	0.159	3.783

*β*, regression coefficient; *SE*, standard error; *OR*, odds ratio; *CI*, confidence interval; *BMI*, body mass index

## Discussion

It has been proposed that the increasing relevance of MALAT1 in controlling many various genes in cells is mainly due to its location in the cell nucleus, in addition to its other functions in controlling numerous known signaling pathways [20]. The involvement of MALAT1 was first recognized since it plays a vital role in non-small cell lung cancer (NSCLC), and it has been demonstrated to influence disease metastasis as well as patient outcomes [12]. Many studies have investigated the potential significance of MALAT1 in the development and progression of BC^21^. High MALAT1 transcript levels were reported in breast tumor tissues in the present study, which is consistent with the findings of previous studies on the potential role of MALAT1 in BC development and progression [22]. Although there was significant dysregulation of the levels of miRs and EMT markers examined, no association was identified with MALAT1 levels in the current study. Targeting MALAT1 by decreasing its level in cells via siMALAT1 efficiently inhibited and decreased the proliferation, migration, and metastasis of BC cells by promoting apoptosis and G1-phase cell cycle arrest [23].

Previous studies have focused on the same concept, one of which has been demonstrated by Xu and colleagues, who defined the ability of MALAT1 to trigger EMT, thereby enhancing in vitro migration and invasion of numerous BC cell lines. MALAT1 has been found to induce EMT via the PI3K/AKT pathway [24]. Another study revealed that MALAT1 induces EMT in BC cells by binding to a protein called FOXO1, and a correlation was also associated with a reduced response to trastuzumab treatment in HER2 + BC cells [25]. MALAT1 overexpression promotes cell migration and invasion by binding to miR-1, which reduces the level of Cdc42, a protein implicated in the EMT process [26]. Furthermore, by employing siMALAT1-mediated inhibition of MALAT1 and, conversely, bringing the MALAT1 overexpression vector into a BC cell line, it was found that MALAT1 exerts its effect through interaction with miR-124, which is related to the suppression of BC progression [27]. Li and others revealed a new mechanism explaining the function of MALAT1 in the control of EMT in BC. These researchers revealed that its transcript exhibited proinflammatory activity and was capable of controlling lipopolysaccharide-induced inflammation and cellular EMT [28]. MALAT1 has been shown to have paradoxical effects on cell growth and invasion in several in vivo experiments in which MALAT1 knockout in human BC cells was shown to confer a metastatic ability that was abolished by MALAT1 re-expression [29]. Meta-analysis indicates that the biological function of MALAT1 may vary depending on tumor subtype, cellular context, and experimental model [30]. Although MALAT1 is widely reported to act as a competing endogenous RNA, its regulatory effects are highly context-dependent and may involve alternative mechanisms such as chromatin remodeling, transcriptional control, or protein interactions rather than direct miRNA sponging in luminal breast cancer.

Recent evidence has highlighted the role of circular RNAs in promoting breast cancer metastasis through well-defined molecular mechanisms. Zeng et al. demonstrated that hsa_circ_0060467 is significantly upregulated in breast cancer liver metastases and contributes to metastatic progression by forming a complex with the RNA-binding protein eIF4A3 while simultaneously acting as a sponge for miR-1205. This interaction resulted in enhanced metastatic potential and aggressive tumor behavior, underscoring the importance of circRNA-mediated regulatory networks in organ-specific metastasis. These findings further support the notion that non-coding RNAs participate in cancer progression through diverse mechanisms beyond linear RNA–miRNA interactions [31]. In another context, a recently published review study indicated that circular RNAs (circRNAs) are emerging regulators of breast cancer metastasis exhibiting dual oncogenic and tumor-suppressive functions depending on cellular context. They influence key metastatic processes, including EMT, angiogenesis, immune evasion, and metabolic adaptation, through several pathways such as PI3K/AKT and STAT3^32^.

Despite the high level of MALAT1 in postmenopausal women and the larger tumor size, as well as the presence of lymph node metastasis, MALAT1 expression did not reach significant values. Our data indicated that a higher MALAT1 level was associated with obesity luminal breast cancer patients. The association between BMI and BC risk has received much attention in the last few years, but the findings are still controversial. In one study, a dose‒response meta-analysis of prospective cohort studies showed that every 5 kg/m^2^ increase in BMI corresponded to a 2% increase in BC risk in women. However, a higher BMI could be a protective factor against breast cancer risk in premenopausal women [33]. MALAT1 is an important lncRNA that plays a relevant role in adipogenesis and obesity and is associated with different diseases, such as NAFLD and liver metabolism [34], osteoarthritis (OA) [35], and type 2 diabetes (T2D) [36]. Our data indicated that a higher level of MALAT1 in obese patients than in non-obese patients might reveal its indirect role in the development and progression of BC.

miR-20a-5p is considered a member of the miR17/92 cluster and is closely associated with different types of cancer [37]_,_ where it plays important roles in various pathways, including proliferation signaling, invasion, metastasis, angiogenesis, the cell cycle and apoptosis [38]. Our research team reported an upregulation of miR-20a-5p in BC tissues compared to normal tissues. Furthermore, this increase was positively correlated with the induction of EMT markers, including SNAI1, Slug, E-Cadherin, and N-Cadherin, confirming its potential function in LBC progression by inducing the EMT process. It has been reported that miR-20a-5p is highly expressed in TNBC tissues and cell lines and that this increase promotes migration and invasion through direct targeting of Runt-related transcription factor 3 (RUNX3) as well as Bin and p21, the downstream targets of RUNX3, suggesting its potential clinical application in TNBC [39]. Another study demonstrated the oncogenic role of miR-20a-5p in BC progression and showed that the PTENP1/miR-20a-5p/PTEN axis is involved in the proliferation and invasion of BC cells via the PI3K/AKT pathway, which might provide a promising therapeutic target for BC^40^. An earlier study demonstrated that the upregulation of miR-20a-5p in BC tissues and in the exosomes of MDA-MB-231 cells enhances the proliferation and differentiation of osteoclasts via targeting SRCIN1, suggesting that exosomes or miR-20a-5p could be new therapeutic targets for BC^41^. In bladder cancer, an oncogenic effect of miR-20a-5p has been confirmed. High expression of miR-20a-5p has been reported in tissues and cell lines to induce EMT progression by inhibiting the suppressive effect of the NR4A3 gene [42]. The overexpression of miR-20a-5p in HCC tissues significantly promoted HCC cell proliferation, migration and invasion and inhibited apoptosis in cell lines. The authors reported that the miR-20a-5p/RUNX3/EMT axis is an important regulatory pathway for inducing EMT and cell migration in HCC cells [43].

To address the role of micR-20a-5p as a prognostic factor in LBC, we attempted to correlate its expression level with some of the clinical data available. The present results indicated an increase in micR-20a-5p accompanied by the presence of lymph nodes, as well as tumor size, in addition to its increase in postmenopausal women, but these correlations were not significant. This is mainly due to differences in ethnicity, the methods used in the laboratory, or the limited number of patients included in this study. Moreover, a significant elevation was associated with both family history and the location of the tumor on the left side compared to the right side. A correlation between the expression levels of a set of 450 miRNAs and different clinicopathological features of breast cancer patients revealed a significant association between higher levels of miR-20a-5p and higher-grade tumors in TNBC patients than in patients with other subtypes, as described in a data analysis study performed on Northern Norway patients [44]. To determine the role of miR-20a-5p in the progression and metastasis of NSCLC, a study was conducted and demonstrated that the level of miR-20a-5p was greater in cancer cells and tissues and was accompanied by large tumor sizes as well as metastasis through the lymphatic system. Its mechanism of action involves direct targeting of the KLF9 gene, which has a distinct and well-known role in both cell proliferation and invasion [45].

Essentially, both MALAT1 and miRs participate in the complex gene regulatory network that governs EMT and drug resistance in cancer and their interaction can have significant consequences for tumor behavior and progression, as well as response to treatment. This study demonstrated that there is no interaction between them, particularly in terms of affecting EMT and, consequently, drug resistance. Only miR-20a-5p had an effect on EMT, independent of MALAT1.

## Conclusion

The present study revealed that MALAT1 plays a significant role in the development and progression of LBC, regardless of its relationship with either miRs or EMT pathways. Furthermore, miR-20a-5p plays an oncogenic role in the development and progression of LBC by acting as an EMT inducer. This study might open up new therapeutic targets for LBC.

### Limitations

This study has some limitations which include: the statistical result to identify the correlations between MALAT1 expression and clinicopathological characteristics may have been affected by the very small sample size. Moreover, the results may not be as applicable to other subtypes of breast cancer as the study population was limited to Egyptian luminal cancer patients. functional assays were not performed to confirm the correlation between MALAT1, miRNAs, and EMT markers. Furthermore, EMT markers were not validated at the protein level, which might not accurately reflect post-transcriptional regulatory effects.

### Future prospective

Future studies should include larger sample size with diverse ethnic backgrounds to validate the clinical relevance of MALAT1 and miR-20a-5p in breast cancer. Functional in vitro and in vivo assays are recommended to elucidate the molecular mechanisms of MALAT1- and miR-20a-5p-mediated EMT regulation. Furthermore, integrating protein-level analyses and exploring circulating ncRNAs as non-invasive biomarkers could enhance translational applicability.

## Data Availability

The datasets used and/or analyzed during the current study are available from the corresponding author on reasonable request.

## Abbreviations

EMT: epithelial- mesenchymal transition
MALAT1: metastasis-associated lung adenocarcinoma transcript 1
miR: microRNA
lncRNAs: long non-coding RNA
NSCLC: non- small cell lung cancer
HER2: human epidermal growth factor 2
ER: Estrogen receptor
PR: progesterone receptor
BMI: body mass index
PI3K/AKT: phosphoinositide 3-kinase/ protein kinase B
RUNX3: Runt-related transcription factor 3
CDC42: cell division cycle 42
qPCR: quantitative polymerase chain reaction
NCBI: national center for biotechnology information
NCI: national cancer institute
ENCORI: encyclopedia of RNA interactomes
